# Analysis of uroseptic shock after ureteroscopy for ureteral calculi during pregnancy: a case report

**DOI:** 10.1186/s12894-023-01299-2

**Published:** 2023-07-27

**Authors:** Wen Tang, Zhifei Xie, Mingwen Liu, ZeJu Zhao, Tao Wu

**Affiliations:** Department of Urology, The Affiliated Hospital of Zunyi Medical University, No. 149 Road Dalian, Huichuan District, Zunyi, China

**Keywords:** Uroseptic shock, Pregnancy, IABP, CRRT, VA-ECMO

## Abstract

**Background:**

Uroseptic shock secondary to ureteral calculi during pregnancy is rare. It is characterized by rapid onset, rapid progression, aggressive disease, limited treatment, poor prognosis, and a mortality rate higher than 20% with improper or delayed management. A clear diagnosis is made based on typical clinical symptoms and abdominal ultrasound, often requiring combined multidisciplinary treatment and the simultaneous release of the obstruction. The high mortality rate is mainly related to inappropriate early treatment of stones and infections or failure to intervene in a timely manner.

**Case presentation:**

A 21-year-old first-time pregnant patient with uroseptic shock was admitted to our intensive care unit. The patient was successfully treated at our hospital with multidisciplinary cooperation, high-dose vasoactive drugs, IABP, CRRT, VA-ECMO, and termination of pregnancy.

**Conclusions:**

Timely relief of obstructions, termination of pregnancy, and the provision of IABP, CRRT, and VA-ECMO when necessary in critically ill patients with uroseptic shock during pregnancy can improve the success rate of resuscitation.

## Background

Sepsis is defined as life-threatening organ dysfunction caused by a dysregulated host response to infection [1]. Maternal sepsis is defined as sepsis during pregnancy or the postpartum period, with pyelonephritis, chorioamnionitis and endometritis being the most common sources of infection [2]. Regarding maternal urosepsis, pyelonephritis due to upper urinary tract obstruction caused by ureteral calculi is the most common source of infection. The incidence of urolithiasis during pregnancy has been reported as 1/1500, similar to that in the nonpregnant population [3]. However, physiological, immunological and mechanical changes during pregnancy make pregnant women more susceptible to infection than nonpregnant women [4]. In addition, physical adaptation to pregnancy may conceal signs and symptoms of infection and sepsis, which may delay diagnosis and treatment [5]. Urosepsis accounts for 5.0–7.0% of sepsis cases, with a mortality rate of 28.3–41.1% [6]. Among pregnant women, 1.81 cases of sepsis per 1000 were diagnosed, 61% and 25% of which originated in the genital tract and urinary tract, respectively [7]. The foetal mortality rate due to bacteraemia in pregnancy is 10–28% [7]. Despite close supervision and standard treatment measures, there is a high mortality rate.

Patients with sepsis combined with multiple severe complications may fail to respond to general therapeutic measures (including mechanical ventilation, hydration, analgesia, and anti-infection therapy). According to the literature, ECMO has been used to treat acute respiratory distress syndrome (ARDS) due to viral infection, pulmonary embolism and amniotic fluid embolism leading to cardiopulmonary arrest in postpartum women [8]. IABP is also often used in combination with ECMO in patients with cardiogenic shock from various causes. We describe the case of a young pregnant woman in the second trimester with combined urosepsis who experienced a series of severe life-threatening complications (septic shock with cardiopulmonary insufficiency, disseminated intravascular coagulation (DIC), multiple organ dysfunction syndrome (MODS)) and foetal death. We finally succeeded in saving the patient by combining VA-ECMO, IABP and CRRT. There is a lack of systematic evidence regarding the safety and efficacy of mechanical circulatory support for maternal uroseptic shock, and we will provide some new insights with this case.

## Case presentation

**Fig. 1:**
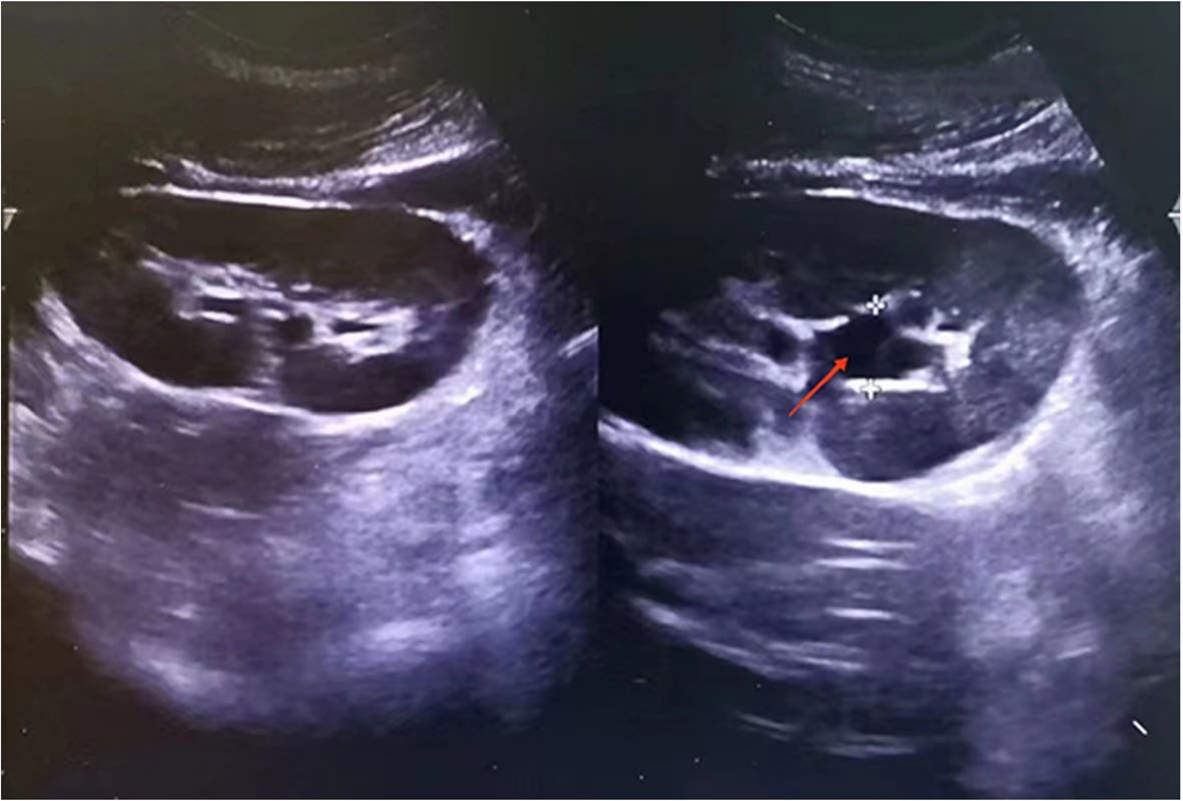
Ultrasound of both kidneys showed separation of the renal pelvis and calyces with a width of approximately 17 mm at the widest point (red arrow)

A 21-year-old female (G1P0) with a monochorionic monoamniotic singleton pregnancy and a BMI of 21.5 kg/m2 had regular obstetric examinations since becoming pregnant, and all foetal indicators were in the normal range. The patient had no previous history of any underlying disease or other medical conditions. At the 12th week after natural conception, the patient began to experience mild, right-sided, low back pain without radiating pain or fever and had stable vital signs; conservative treatment was recommended after consultation at the local hospital. At the 20th week, the patient had recurrent right-sided lumbago, chills, and high fever, so she visited the local hospital again and was diagnosed with right ureteral calculi with hydronephrosis and urinary tract infection after urological ultrasound (Fig. 1). An obstetric ultrasound showed the following: a mid-uterine pregnancy, a single live foetus, a foetal biparietal diameter of 50 mm, a head circumference of 173 mm, an amniotic fluid depth of 40 mm, and a heart rate of 148 bpm. The above symptoms were treated with intravenous magnesium sulphate (MgSO4), imipenem cystatin anti-infection therapy, symptomatic hypothermia, etc. The above symptoms did not improve, so ureteroscopic drainage with an F5 double “J” tube was performed immediately under local anaesthesia. After the operation, the patient’s back pain was relieved, but she developed irritability, persistent hyperthermia, an accelerated heart rate, and decreased blood pressure. Uroseptic shock was considered, and respiratory distress worsened after 18 h of resuscitation. She was urgently transferred to the hospital after tracheal intubation. Physical examination showed the following: a temperature of 35.5℃, a pulse of 127 bpm, a breathing rate of 18 bpm (ventilator-assisted breathing), an oxygen saturation rate of 100%, and deep coma, with a Glasgow Coma Scale (GCS) score of 1/1/T; blood pressure was not measured. Both pupils had diminished light reflexes, petechiae were scattered on the skin, the abdomen was bulging; the patient had clear uterine contours, no tension or pressure pain, no contractions, a uterine height of 28 cm, bilateral lower extremity oedema, and no catheter. The foetus was in an ROA position, and the foetal heart rate was 0 bpm. Laboratory tests showed the following: a WBC count of 19.58*10^9^/L, an N count of 0.85%, a CRP level of 31.10 mg/L, an ALT level of 1240 U/L, an AST level of 5863 U/L, an Scr level of 110 µmol/L, a PT of 35.20 s, an APTT of 135.50 s, a Tn level of 712.90 ng/L, and a urinary WBC count of 761 cells/µL. Echocardiography showed reduced motion in all phases of the left ventricular wall and a left ventricular EF of 18–24%. Obstetric ultrasound showed a midterm, singleton pregnancy and foetal death. Ultrasound of the thoracic and abdominal cavities showed large bilateral effusions in the thoracic and abdominal cavities; urological ultrasound showed no significant abnormalities in the sonogram of either kidney. The preliminary diagnosis was uroseptic shock, DIC, and MODS.

## Resuscitation process

**Fig. 2:**
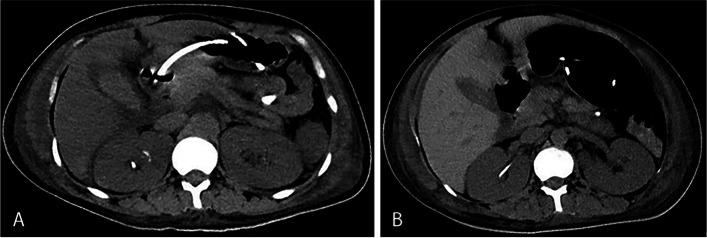
Computed tomography scan of both kidneys. **A** Bilateral renal and perirenal conditions at the first week after admission. **B** Bilateral renal and perirenal conditions at the third week after admission

A central venous catheter was immediately placed for fluid infusion and blood transfusion; norepinephrine (32 mg), epinephrine (10 mg), posterior pituitary hormone (28 U), antibiotics, hormones, and proton pump blockers were pumped continuously. The catheter was retained for urine volume conservation, blood and urine sample cultures, dynamic blood gas monitoring, biochemical indicators, and other treatments. After transfer to the ICU, the blood pressure failed to maintain a normal range, and CRRT was provided to clear inflammatory mediators. Due to respiratory and circulatory failure, after multidisciplinary discussion, it was decided to use VA-ECMO to provide extracorporeal respiration and circulation and reduce cardiac energy consumption to help increase the cardiac output. IABP raises the aortic diastolic pressure and increases coronary perfusion to improve myocardial oxygen supply. After 24 h of treatment with the above measures, the patient’s body temperature did not rise, her blood pressure fluctuated in the normal range, she had systemic ecchymosis, anuria, and severe coagulation dysfunction, and her absolute values of blood WBCs and neutrophils were further increased. Considering the foetal death in utero, immediate ultrasound monitoring was performed with foetal destruction and uterine tissue aspiration. Postoperative placental pathology indicated mild acute amnion-chorioamnionitis. Further autopsy was proposed to determine the cause of foetal death, but the patient’s family refused. For thoracic and abdominal effusions, thoracic and abdominal drainage tubes were placed, and albumin was given to increase the plasma colloid osmotic pressure. After 48 h, the body temperature rebounded, the urine output increased, petechiae did not worsen, and the condition gradually stabilized. Repeat laboratory tests showed the following: a WBC count of 12.19*10^9^/L, a N count of 0.91%, a CRP level of 22.50 mg/L, an ALT level of 128 U/L, an AST level of 539 U/L, a Scr level of 118 µmol/L, a PT of 13.80 s, an APTT of 36.10 s, and a Tn level of 265.70 ng/L. Echocardiography showed no significant abnormalities in the motion of the segments of the left ventricular wall and a left ventricular EF of 43–58%. IABP and VA-ECMO were gradually withdrawn, the dosage of vasoactive drugs was gradually reduced, and gastrointestinal nutrition was added. After 1 week, consciousness gradually returned, and the patient could move her limbs moderately and independently, but fever reappeared. Blood culture indicated multidrug-resistant bacteria and fungal infection. After treatment according to drug sensitivity and increased rehabilitation measures, the patient’s condition was stabilized again, consciousness improved, and the GCS score was 9. Repeat computed tomography (CT) examination at the first and third weeks showed no abnormalities in the size, morphology or density of either kidney, and the perinephric fluid was gradually absorbed (Fig. 2). She was discharged from the hospital after being referred to the Plastic Surgery Department for debridement and flap repair for localized skin necrosis in the left inguinal region, which was caused by a left femoral artery fistula after ECMO. At the 1-year follow-up, the patient declared that she could take care of herself, had no significant complications, and planned to have another pregnancy.

## Discussion and conclusions

Urosepsis in pregnancy is a rare condition that poses a serious threat to the lives of the mother and child. Maternal sepsis is a major cause of maternal morbidity and mortality and an important cause of premature foetal delivery or foetal loss [5]. The urinary tract undergoes some anatomical and functional changes during pregnancy, leading to the development of various urinary tract symptoms. Urinary tract infections and urolithiasis during pregnancy are not uncommon and are associated with miscarriage, preterm delivery and foetal growth restriction [9–11]. Regarding the imaging diagnosis of urinary stones, ultrasound is the first-line imaging technique in pregnant women. However, the sensitivity of ultrasound for ureteral stones in this population is only 34–86% [12]. In circumstances where an ultrasound demonstrates hydronephrosis but fails to demonstrate an obstructing ureteral stone, urologists may hesitate to order a CT scan due to concern of foetal radiation [13]. The radiation dose from a single CT study is very small, and the risk to the foetus is very low. As such, the American College of Radiology (ACR) and the American College of Obstetrics and Gynecology (ACOG) support the use of abdominopelvic CT for suspected nephrolithiasis if medically necessary, even in the first trimester [14]. In scenarios where the patient is clinically worsening and the diagnosis of renal colic needs to be determined with certainty, CT should be considered as the next diagnostic test [13].

The most common indication for urological surgery in pregnant patients is symptomatic urolithiasis [11, 12, 15]. Patients requiring more aggressive treatment include those with refractory pain due to stones, bilateral obstruction, single kidney obstruction, and urinary tract infection or sepsis [12]. Conservative treatment of ureteral stones during pregnancy without definite infection can benefit 70–80% of patients [9, 16, 17]. If conservative treatment fails, the development of infection due to hydronephrosis, especially in cases of impaired renal function, and sepsis is considered a contraindication to conservative treatment, and immediate surgical intervention should be performed [11, 18]. Advances in endourology have improved the safety and efficacy of the ureteroscopic management of stones during pregnancy [12, 15]. Some patients without infection and those with small stones, spacious ureteral lumen, small distortion, and stable pregnancy can undergo first-stage ureteroscopy lithotripsy [19–21]. The results of the Cormier et al. study suggest that ureteroscopic drain placement is a safe option for patients with unresolved renal colic during pregnancy and that percutaneous nephrostomy can be used for rapid decompression of the renal cavity in patients who have developed sepsis [18]. Butticè et al. demonstrated that the timing of surgery is the most important factor affecting the risk of sepsis and the threat of abortion, that the duration of surgery is a major risk factor for premature uterine contractions (PUCs) and that delaying surgery may also lead to an increased incidence of PUCs and preterm delivery by increasing the risk of sepsis for the mother and foetus [22]. Therefore, both early recognition and timely intervention are necessary steps to reduce maternal morbidity and mortality from sepsis and to reduce the risk of PUCs [5, 22].

Primary uroseptic shock from ureteral stones during pregnancy is relatively rare, and timely nonsurgical treatment can mostly create surgical opportunities; ureteral stenting or percutaneous nephrolithotomy is currently the most commonly used treatment [10, 23, 24]. Indecision or inappropriate measures will accelerate the progression of the disease, and mortality will increase significantly. In this case, the patient had obvious preoperative symptoms and systemic inflammatory reaction syndrome (SIRS). Urosepsis developed, and stable vital signs, intraureteral stent drainage under rehydration and antibiotic application helped to relieve the condition. Unfortunately, the condition worsened after surgery and soon progressed to shock. The possibility of a high intraoperative ureteroscopy flushing pressure, a prolonged operation time, and large amounts of bacteria and toxins entering the blood in a short period of time cannot be ruled out. Postoperative failure to leave a catheter in place or poor management of the catheter leads to incomplete obstruction relief, and urinary reflux increases the continued entry of bacteria and toxins into the blood, driving the progression of the disease [25, 26].

Standardized treatment within 6 h of uroseptic shock is important [27]. According to Whiles et al., each hour until initial antimicrobial administration was associated with an 8.0% increase in the risk of progression to septic shock [28]. The patient failed to receive effective treatment during this golden hour, did not receive timely consultation or referral and was referred after 18 h of shock, significantly delaying resuscitation and resulting in rapid deterioration of her condition. In the face of a patient with prolonged uraemic septic shock, involuntary respiration, an undetectable blood pressure, intrauterine stillbirth and multiple system failure, the combined treatment team quickly established central venous access, adjusted rehydration, provided antihypertensive drugs according to the central venous pressure, judged the patient’s condition and formulated a plan based on cardiac ultrasound and biochemical and blood gas indices. In response to the effects of heart failure, SIRS, and ARDS on the patient, IABP, CRRT, and VA-ECMO were applied in combination, and resuscitation measures such as ensuring unobstructed catheter drainage and timely termination of pregnancy were also taken at the same time. The patient was finally stabilized 48 h after admission. Extracorporeal life support therapy has been proven to be safe in select nonpregnant patients [29]. Pregnancy is not a contraindication to any supportive therapy, and there are numerous reports of good maternal and foetal outcomes in pregnant women who receive ECMO support for respiratory failure during pregnancy. However, due to the significant risks inherent in ECMO, its safety and efficacy have not been demonstrated in different types of pregnant patients [8, 30]. In this case, the patient was in respiratory and circulatory failure due to uroseptic shock, and we preferred V-A ECMO combined with IABP for cardiopulmonary support. We considered the combination of IABP- and VA-ECMO-assisted cardiac and pulmonary work, reduced energy consumption, increased coronary blood supply and improved myocardial function, effectively safeguarding circulatory dynamics and oxygenation, increasing oxygen delivery and improving tissue perfusion [31, 32]. CRRT assisted the kidneys, excreted excess water, metabolic waste and inflammatory factors in a timely and effective manner, blocked the ongoing damage to the heart, lungs and kidneys from toxins and inflammatory factors, and lay the foundation for antibiotics to work effectively [33, 34]. Second, high-quality care was also very important, especially respiratory management, decubitus prevention, and deep vein thrombosis care [35].

In summary, most patients with ureteral stones in pregnancy can obtain significant improvement in their symptoms after conservative treatment, but the condition of such patients can deteriorate rapidly, and those with low back pain and fever need to be on high alert once the treatment measures are determined to be inappropriate because uroseptic shock can quickly develop. Sepsis is a “time-dependent” pathological condition [2], and early diagnosis and timely intervention can significantly improve maternal and foetal prognoses; however, the normal physiological changes during pregnancy may make early diagnosis a challenge. Interventions focus on the identification of the source of infection, the correction of hypotension, the use of high-grade antibiotics, and timely surgical intervention. Unfortunately, there is still a small percentage of patients whose conditions are exacerbated; these patients enter the septic shock decompensation phase, at which point conventional treatment measures are no longer helpful. We believe that providing timely multidisciplinary combination therapy with IABP, CRRT and V-A ECMO to assist in improving vital organ function and clearing inflammatory factors and toxins as early as possible, if necessary, can improve the salvage success rate. With this case, we demonstrated the safety and efficacy of combined IABP, CRRT, and V-A ECMO in a pregnant patient with uroseptic shock, providing a new basis for extracorporeal life support during pregnancy.

## Data Availability

The datasets used and/or analysed during the current study are available from the corresponding author upon reasonable request.

## Abbreviations

IABP: Intra-Aortic Balloon Pump
CRRT: Continuous Renal Replacement Therapy
VA-ECMO: Venous-Arterial Extracorporeal Membrane Oxygenation
GCS: Glasgow Coma Scale
bpm: Beats per minute
WBC: Write blood cell
N: Neutrophil
CRP: C-reaction protein
ALT: Alanine aminotransferase
AST: Aspartate aminotransferase
Scr: Serum creatinine
PT: Prothrombin time
APTT: Activated partial thromboplastin time
Tn: Troponin
EF: Ejection fraction
ROA: Right occiput anterior
DIC: Disseminated intravascular coagulation
MODS: Multiple organ dysfunction syndrome
ICU: Intensive care unit
CT: Computed tomography
SIRS: Systemic inflammatory reaction syndrome
ARDS: Acute respiratory distress syndrome
ACR: American College of Radiology
ACOG: American College of Obstetrics and Gynecology
PUCs: Premature Uterine Contractions
